# A Critical Review of Zebrafish Models of Parkinson’s Disease

**DOI:** 10.3389/fphar.2022.835827

**Published:** 2022-03-15

**Authors:** Jillian M. Doyle, Roger P. Croll

**Affiliations:** Department of Physiology and Biophysics, Faculty of Medicine, Dalhousie University, Halifax, NS, Canada

**Keywords:** zebrafish (brachydanio rerio), parkinson’s disease (PD), animal models, neurodegeneration, dopamine

## Abstract

A wide variety of human diseases have been modelled in zebrafish, including various types of cancer, cardiovascular diseases and neurodegenerative diseases like Alzheimer’s and Parkinson’s. Recent reviews have summarized the currently available zebrafish models of Parkinson’s Disease, which include gene-based, chemically induced and chemogenetic ablation models. The present review updates the literature, critically evaluates each of the available models of Parkinson’s Disease in zebrafish and compares them with similar models in invertebrates and mammals to determine their advantages and disadvantages. We examine gene-based models, including ones linked to Early-Onset Parkinson’s Disease: *PARKIN, PINK1, DJ-1*, and *SNCA*; but we also examine *LRRK2,* which is linked to Late-Onset Parkinson’s Disease. We evaluate chemically induced models like MPTP, 6-OHDA, rotenone and paraquat, as well as chemogenetic ablation models like metronidazole-nitroreductase. The article also reviews the unique advantages of zebrafish, including the abundance of behavioural assays available to researchers and the efficiency of high-throughput screens. This offers a rare opportunity for assessing the potential therapeutic efficacy of pharmacological interventions. Zebrafish also are very amenable to genetic manipulation using a wide variety of techniques, which can be combined with an array of advanced microscopic imaging methods to enable *in vivo* visualization of cells and tissue. Taken together, these factors place zebrafish on the forefront of research as a versatile model for investigating disease states. The end goal of this review is to determine the benefits of using zebrafish in comparison to utilising other animals and to consider the limitations of zebrafish for investigating human disease.

## 1 Introduction

### 1.1 Parkinson’s Disease

Parkinson’s Disease (PD) is the second most common neurodegenerative disorder after Alzheimer’s Disease. It typically affects individuals over the age of 65, although Early Onset Parkinson’s Disease (EOPD) is well-noted. With the current aging world population, the number of people living with PD is expected to reach 12 million by the year 2040 (Dorsey and Bloem, 2018; Dorsey et al., 2018). Symptoms primarily affect movement, including tremors, bradykinesia (slow movements), rigidity and postural instability; in addition, patients may also manifest cognitive symptoms like impaired memory and executive dysfunction (Rana et al., 2015). Despite extensive research, no definitive cause has been identified. Numerous genes have been implicated, including, but not limited to: *LRRK2, SNCA* (*PARK1/4*), *DJ-1, PINK1,* and *PARKIN* (Best and Alderton, 2008). The majority of PD cases, however, are sporadic and not associated with any particular gene (Lang and Lozano, 1998; Coulom and Birman, 2004). Environmental factors, like chemical exposure to MPTP, pesticides and solvents, may also play a role in the onset of PD (Best and Alderton, 2008; Vázquez-Vélez and Zoghbi, 2021).

The two main physiological characteristics of PD are the loss of nigrostriatal dopaminergic neurons and diffuse brain deposition of Lewy bodies, proteinaceous inclusions mainly containing α-synuclein fibrils. (Braak and Braak, 2000; Davie, 2008). Many interconnected factors contribute to the pathophysiology of PD, some of which are not fully elucidated (Vázquez-Vélez and Zoghbi, 2021).

Alpha-synuclein, produced by the *SNCA* gene, has been associated with several linked cellular pathways that are altered in PD pathophysiology. Impairments in lysosomal pathways, whether genetically induced or not, cause a reduction in α-synuclein degradation (Cuervo et al., 2004; Vogiatzi et al., 2008; Vázquez-Vélez and Zoghbi, 2021). Dysregulation and/or improper processing of α-synuclein also leads to mitochondrial dysfunction and the generation of reactive oxygen species (ROS). Although the mechanisms are not precisely clear, mitochondrial dysfunction can also affect α-synuclein in a feedback loop (Nakamura et al., 2011; Di Maio et al., 2016).

Mitochondrial dysfunction itself is a fundamental part of PD pathophysiology and is an important factor in dopaminergic cell death (Malpartida et al., 2021). The genes *PINK1* and *PARKIN* have been linked to mitophagy and recent studies suggest that mutations in the genes *LRRK2* and *SNCA* also contribute to mitochondrial dysfunction (Malpartida et al., 2021). Several of the chemical substances identified as environmental risk factors for developing PD specifically target Complex I of the mitochondrial electron transport chain (ETC) and subsequently cause neuron death (Moore et al., 2005).

ROS also play a large role in PD pathophysiology and are tightly connected with dysregulation of α-synuclein and mitochondrial dysfunction (Weng et al., 2018). Alpha-synuclein promotes pro-inflammatory factors in microglia, the immune cells of the central nervous system, which results in ROS production (Block et al., 2007; Thameem Dheen et al., 2007). Mitochondrial dysfunction leads to production of ROS which then interferes with the ETC and leads to a reduction in cellular energy stores, and subsequently causes cell death (Bhat et al., 2015; Hang et al., 2015). Even dopamine, the neurotransmitter at the heart of PD, is notably unstable and can auto-oxidize and form ROS, in addition to ROS being generated as by-products of dopamine degradation by monoamine oxidase B (MAO-B) (Youdim et al., 2006; Hastings, 2009). Many other elements have been implicated in PD; however a full review of the disease pathophysiology is beyond the scope of this article. For a more in-depth treatment, the reader is referred to excellent reviews on the topic (Moore et al., 2005; Weng et al., 2018; Malpartida et al., 2021; Vázquez-Vélez and Zoghbi, 2021).

Due to the myriad of mechanisms and potential causes involved in PD, the development of a model recapitulating the main features of the disease, including the selective degeneration of nigrostriatal neurons, Lewy bodies-like pathology and motor deficits has proven to be problematic.

### 1.2 Using Animal Models to Investigate Causes

Studying *in vitro* human cell models can impart substantial information, but such models cannot yet replicate the multi-system interactions observed in the human disease or *in vivo* animal models. When choosing a model organism, a critical analysis must be performed to determine the value of findings with respect to human disease. Most importantly, studies of a model must possess a high level of replicability (Caramillo and Echevarria, 2017). A meta-analysis of transcriptional profiles of transgenic mouse models of Alzheimer’s Disease found that those models were not always similar within their own group, between groups or to human Alzheimer’s Disease models, indicating poor replicability and it is expected that this finding would also be relevant to models of PD (Hargis and Blalock, 2017). Accordingly, many of the therapies developed with animal models do not translate well to human clinical trials for a host of reasons (van der Worp et al., 2010). These challenges must be addressed when developing animal models of human disease.

Mice and rats are traditional animal models for studying human diseases (Jucker, 2010). As mammals, they possess relatively similar anatomy and physiology to humans; and in the case of mice, numerous transgenic strains have been developed. However, despite their small size, mice are still expensive to keep in large numbers, and transgenic strains are labour-intensive to produce (Jucker, 2010). Conversely, invertebrates like the fruit fly, *Drosophila melanogaster*, and the nematode, *Caenorhabditis elegans,* have been used for many years as medical models. Such invertebrates possess many of the same basic cellular processes and basic gene functions as mammals but are inexpensive and suitable for large scale experiments (Ségalat, 2007). The obvious drawback of these invertebrates is that they lack similar brain structures and organ systems to humans, so they may not model human disease accurately. The zebrafish has become widely established as a medical model in recent years, conveniently combining the versatility of invertebrates with the anatomical similarity of mammals (Spence et al., 2008).

### 1.3 Benefits of Using Zebrafish

Zebrafish have been used to study numerous human diseases, including neuropsychiatric and neurodegenerative diseases, owing to similarities with human brain physiology and anatomy (Xi et al., 2011; Fontana et al., 2018). To date, models have been developed for some of these diseases including amyotrophic lateral sclerosis (ALS) and Huntington’s Disease, with the bulk of extant research focused on Alzheimer’s and Parkinson’s Diseases (Xi et al., 2011; Babin et al., 2014; Martín-Jiménez et al., 2015). The zebrafish brain is comparable to the mammalian brain, with fore-, mid- and hindbrain sections also containing a diencephalon and telencephalon and primary neurotransmitter function is generally similar to that in humans (Panula et al., 2006; Lieschke and Currie, 2007; Toledo-Ibarra et al., 2013; Stoyek et al., 2015; Caramillo and Echevarria, 2017). Like mammals, zebrafish possess a blood brain barrier, and permeability tests indicate that its physiological properties are conserved between zebrafish and humans (Cuoghi and Mola, 2007; Wager and Russell, 2013). In addition to their physiological benefits, zebrafish also exhibit sophisticated cognitive behaviours, such as learning and retaining associations, and they manifest well-documented anxiety behaviours (Lieschke and Currie, 2007).

One of the key advantages of zebrafish is their suitability for genetic manipulation, which has led to the development of thousands of mutant, transgenic and otherwise genetically-altered strains (See Section 2.1) (Ruzicka et al., 2019). For example, adult zebrafish of some mutant strains are optically transparent, enabling *in vivo* imaging of internal tissues and cells (White et al., 2008). These transparent mutations extend the optical transparency that is already a useful feature of all larval zebrafish into adulthood (Parichy et al., 2009). Genetically altered zebrafish featuring a genotype or phenotype of interest can be produced with less effort than their rodent counterparts, as DNA or RNA can more easily be injected at the single cell stage, due to external fertilisation and development of the fish (Clark et al., 2011). Fluorescent reporters linked to specific promotors provide an array of imaging opportunities (Halpern et al., 2008). Besides traditional reporters, like green fluorescent protein (GFP), genetically encoded calcium indicators like GCaMP and the optogenetic reporter channel rhodopsin can easily be incorporated into transgenic zebrafish lines (Halpern et al., 2008; Howe et al., 2017). Combining the ability to create custom transgenic lines with current imaging techniques presents a unique opportunity to study the functions of genes in a living animal, something not easily achieved with other models. With advanced microscopy techniques imaging live transgenic lines is becoming more accessible. Light sheet microscopy enables the user to illuminate and image an entire plane of tissue, which has advantages over traditional point-scanning methods like confocal and two-photon (Hillman et al., 2019). Fluorescence lifetime imaging microscopy (FLIM) offers the ability to perform *in vivo* observations of zebrafish over the temporal lifespan of the fluorophore, and can provide additional data on overlapping emission spectra and the intensity of the fluorophore (Zhang et al., 2021). In addition to the advantages described above, zebrafish are becoming increasingly popular for use in high-throughput screens due to their prolific reproduction and cost-efficient size. Based on these factors, potential therapeutics and genes can be quickly identified in a fraction of the time required for rodents (See Section 5: Discussion).

### 1.4 Dopaminergic Neurons in Zebrafish

The dopaminergic system, the primary site of PD, has been extensively studied in zebrafish. Retrograde tracing studies in the adult zebrafish brain found that dopaminergic neurons projecting to the ventral telencephalon are located in the posterior tuberculum of the ventral diencephalon (Xi et al., 2011). Although it has been suggested that these dopaminergic neurons in the ventral diencephalon may be analogous to the midbrain dopaminergic neurons of the nigrostriatal pathway, this has been questioned by others (Wullimann and Rink, 2001; Tay et al., 2011). The dopaminergic system in embryonic zebrafish is also well characterized. Dopaminergic neurons are first detected at 18 h post fertilization (hpf) in a cluster in the ventral diencephalon, and by 72 hpf, the organization of the central nervous system is complete and subsequent development only adds increased numbers of neurons (Kimmel et al., 1995; Wullimann and Rink, 2001). In addition to the ventral diencephalon, dopaminergic neurons are also found in the olfactory bulb, preoptic region, retina and pretectum (Rink and Wullimann, 2002). Zebrafish had been found to have similar dopaminergic signalling pathways to mammals, and transcription factors have been shown to play evolutionarily conserved roles in the development of zebrafish dopaminergic neurons (Xi et al., 2011). Dopaminergic neurons in zebrafish are sensitive to oxidative stress, which is one of the main causes of their death in PD (McCormack et al., 2006; Rappold et al., 2011; also see above). The well-characterized dopaminergic system, along with the other benefits mentioned above, make the zebrafish a suitable model for studying PD.

### 1.5 Goals of This Review

Reviews published in the last few years have catalogued the numerous gene and chemically based models of PD in zebrafish (Razali et al., 2021; Wang et al., 2021). However, it is still unclear how these models compare to PD models in more established animals, like rodents, *Drosophila* and *C. elegans*. This paper presents a critical review of several gene-based and chemical-based models of PD in zebrafish. Each of the currently available models will be evaluated on its strengths, weaknesses and contributions, or potential future contributions, to knowledge of the disease; however, emphasis will be placed on the most characterized and relevant models.

## 2 Gene-Based Models

### 2.1 Creating Gene-Based Models in Zebrafish

The models described in this section use various methods of genomic manipulation, each with its own advantages and disadvantages.

One of the earliest methods of creating models was to expose zebrafish to mutagenic substances and then perform extensive phenotyping to identify fish with mutations of interest (Driever et al., 1996; van Eeden et al., 1998). This labour intensive process was facilitated by the advent of TILLING (targeting induced local lesions in genomes) which can easily identify genes of interest (Amsterdam and Hopkins, 2006; Rafferty and Quinn, 2018). Another method for disabling genes is using zinc-finger nucleases (ZFNs), restriction enzymes which cleave a target section of DNA. ZFNs are very accurate at targeting specific nucleotide sequences, but sometimes mutations occur at the repair site. Furthermore, this process is expensive and can be difficult to design (Carroll, 2011; Hruscha et al., 2013).

In the past, one of the most popular tools for genetic manipulation had been the transient gene knock down using morpholino oligonucleotides (MOs). MOs work by either blocking translation of target mRNA or preventing splicing of pre-mRNA. This method revolutionized gene manipulation, as it was cost effective and relatively easy to use, with many of the studies reviewed here employing this method. It is well documented that MOs may inhibit off-target genes in addition to, or instead of, the target gene (Bill et al., 2009; Blum et al., 2015). Furthermore, it is difficult to inject precise and reproducible volumes of MOs into zebrafish eggs (Eisen and Smith, 2008).

Many of the gene-based models in this review utilised genetically altered strains of zebrafish, which were created using several methods. In one method, a plasmid containing a gene or nucleotide sequence of interest is injected into zebrafish eggs, and this DNA is eventually integrated into the genome. However, this method has a low rate of success, as only about 5% of the resultant fish possess the desired genotype (Kawakami, 2007). A notable improvement uses the *tol2* system which depends on identified *tol2* sequences, which are found throughout zebrafish DNA. Injected transposase mRNA cleaves the DNA at these sequences, and a plasmid containing a *tol2* construct with desired new DNA inserts into the resulting gap. This method is much more accurate and 50–70% of the offspring inherit the inserted DNA.

The advent of new genomic editing techniques presents the opportunity to create knock downs with fewer off-target effects. One of the most accurate is TALEN (transcription activator-like effector nucleases), which utilises TALEs (transcription activator-like effectors) designed to bind to specific DNA sequences (Joung and Sander, 2013). TALEs are combined with nucleases, which enable them to cleave DNA at precise locations. This method can be used to excise a gene or generate a site for a gene to be inserted. TALEs are relatively easy to design but have labour-intensive cloning steps and may not be accessible to all labs (Hruscha et al., 2013). The newest method is the CRISPR (clustered, regularly interspaced, short palindromic repeats)/Cas9 system, which was developed from a bacterial immune defence mechanism. Emmanuelle Charpentier and Jennifer Doudna won the Nobel Prize in Chemistry in 2020 for their work on CRISPR and it has rapidly become one of the most widely used genetic editing techniques, generally and specifically in zebrafish (Westermann et al., 2021). The first step is the construction of a short piece of synthetic RNA that targets a complementary segment of desired DNA. The Cas9 protein, an enzyme that cleaves DNA, uses the synthetic RNA to identify the site of DNA cleavage (Ran et al., 2013). Like TALEN, it can be used to excise genes or introduce a new gene. CRISPR/Cas9 is cheaper and much less time consuming than either ZFN or TALEN. In zebrafish, CRISPR/Cas9 is highly effective: the mutation can be induced in 86% percent of fish on average and be completely heritable (Hruscha et al., 2013). This technique shows immense promise for the creation of new zebrafish lines.

For a summary of the following specific models refer to Table 1.

**TABLE 1:** Gene-based zebrafish models of Parkinson’s Disease.

Human gene	Zebrafish gene	Study	Adults/Larvae	Method	Dopamine	Results
DJ-1	dj-1	Baulac et al. (2009)	Larvae	MO knock down of *dj-1*	Increased sensitivity of DNs to oxidative stress	Dysregulation in proteins related to mitophagy, redox, stress and inflammation
		Edson et al. (2019)	Adults	CRISPR/Cas 9 removal of *dj-1*	Loss of TH-positive cells	
PINK1	pink1	Anichtchik et al. (2008)	Larvae	MO knock down of *pink1*	Loss of dopaminergic neurons	Severe phenotype
		Sallinen et al. (2009)	Larvae	MO knock down of *pink1*	Increased sensitivity of DNs to MPTP
		Xi et al. (2010)	Larvae	MO knock down of *pink1*	Disruption of DN organization	Decreased swimming, reduced startle response, reduced mitochondria
		Flinn et al. (2013)	Larvae/Adults	*pink1* ^−/−^ mutant (identified with TILLING)	Loss of DNs, ↓ mitochondrial activity	Upregulation of TigarB (MO knockdown rescued *pink1* ^−/−^ effects)
PARKIN	parkin	Flinn et al. (2009)	Larvae	MO knock down of *parkin*	Loss of DN, increased sensitivity	Decreased mitochondrial activity
		Fett et al. (2010)	Larvae	MO knock down of *parkin*	Increased sensitivity	
LRRK2	lrrk2	Sheng et al. (2010)	Larvae	MO knock down *lrrrk2*	Loss of TH-positive cells	Severe lethality
		Sheng et al. (2010)	Larvae	MO knock down of *lrrk2* (WD40)	Loss of DN neurons	Reduced swimming activity
		Ren et al. (2011)	Larvae	MO knock down of *lrrk2* (WD40)	No loss of DNs	No swimming deficits
		Prabhudesai et al. (2016)	Larvae	MO knock down of *lrrk2* (50%)	Loss of DNs	Moderate defects, β-synuclein aggregates
SNCA	β- and γ_1_-synuclein	Milanese et al. (2012)	Larvae	MO knock down of *β* and/or *γ-synuclein*	Alters DN development	Impaired motor functions, rescued by human α-synuclein
	α-synuclein (human)	Prabhudesai et al. (2012)	Larvae	Human *α-synuclein* overexpression		α-synuclein aggregates, apoptosis, rescued by CLR01
	γ_1_-synuclein	Lulla et al. (2016)	Larvae	*γ*_*1*_ *-synuclein* overexpression		γ_1_-synuclein aggregates, rescued by CLR01

*MO, Morpholino oligonucleotides DNs, Dopaminergic neurons TH, Tyrosine hydroxylase.

### 2.2 Parkin

Mutations in the *Parkin* gene are the most common autosomal-recessive mutations in EOPD (Kitada et al., 1998). The *Parkin* gene encodes an E3 ubiquitin ligase which is involved in the proteasome degradation system and also may be involved in mitochondrial function along with the other PD-related genes *PINK1* and *DJ-1* (Flinn et al., 2009). The ParkinQ311X mouse, which expresses a human parkin variant, exhibited dysfunction and degeneration in dopaminergic neurons (Regoni et al., 2020, 2021). However, *Parkin*-null mice showed no evidence of dopaminergic neuron loss, decreased mitochondrial function or abnormal behaviour (Dawson et al., 2010). Conversely, loss of *Parkin* function in *Drosophila* results in decreased numbers of dopaminergic neurons and a reduction of mitochondria in the indirect flight muscles (Greene et al., 2003; Whitworth et al., 2005).

Zebrafish parkin has 62% similarity with the human counterpart and is expressed ubiquitously in larval and adult fish (Flinn et al., 2009). An MO knock down of *parkin* caused a 20% decrease in the numbers of diencephalic dopaminergic neurons, and an increased sensitivity of these neurons to the neurotoxin MPP+ (See Section 3.1) at 3 days post fertilization (dpf) (Flinn et al., 2009). The knock down did not have abnormal mitochondria morphology but did have reduced mitochondrial Complex I activity, something also noted in human patients with *Parkin* mutations (Durcan and Fon, 2015). There was no effect on the normal onset of swimming behaviour at 5 dpf, but it is unclear whether the observed 20% neuron loss would have been sufficient to cause such motor symptoms.

In contrast, another MO knock down of *parkin* in zebrafish showed no loss of dopaminergic neurons but did show increased susceptibility to stress-induced cell death (Fett et al., 2010). There was no effect on mitochondrial morphology, but *parkin* was transcriptionally upregulated in response to mitochondrial stress, as in humans. *Parkin* may also have a protective effect because overexpression inhibits proteotoxic stress, which causes cell death.

Flinn et al. (2009) stated that their MO knock down of *parkin* has biochemical and pathological changes analogous to those in humans with *Parkin* mutations. However, as with other MO knock downs, there is a definite discrepancy between the results in these studies, which may indicate different off-target effects or different knock down efficiencies. Both Flinn et al. (2009) and Fett et al. (2010) indicate that *parkin* has similar protective functions, and they are comparable to the effects seen in humans. A *parkin*-deficient fish created using a more consistent method is required to clarify these results.

### 2.3 PINK1

Mutations in the gene encoding PINK1 (PTEN (phosphatase/tensin homolog)-induced putative kinase I) are the second most common cause of autosomal-recessive EOPD (Xi et al., 2011). Loss of *PINK1* function in humans causes increased lipid peroxidation and decreased function of mitochondrial Complex I (Exner et al., 2007). In *PINK1*-deficient mouse models, no dopaminergic neuron loss was characterized, but dopamine release was impaired (Kitada et al., 2007; Zhou et al., 2007). In *Drosophila*, deactivation of *PINK1* caused various effects, notably death of dopaminergic neurons and muscle degeneration (Yang et al., 2006).

Zebrafish pink1*,* which is 54% similar to human PINK1, is expressed ubiquitously in larvae but found only in the periventricular zones and in some diencephalic dopaminergic neurons of the adult fish brain (Anichtchik et al., 2008). An MO knock down of *pink1* reduced the number of dopaminergic neurons by 40% in the ventral diencephalon and was accompanied by severe defects in body morphology. This severe phenotype was partially rescued by wild-type human PINK1 but not mutant PINK1. There were also differences in mitochondrial function, including increased caspase-3 activity and ROS levels (Anichtchik et al., 2008).

A different MO knock down of *pink1* (Sallinen et al., 2010) was not able to replicate the results from Anichtchik et al. (2008). The severe morphological effects seen in Anichtchik et al. were only noted in the Sallinen et al. study in MOs with strong off-target effects that could not be rescued by *pink1* mRNA. It is likely that the MO developed by Anichtchik et al. may have had strong off-target effects and this knock down should be replicated using a different genetic editing technique. Sallinen et al. (2010) noted no loss of dopaminergic neurons in their knock down, but they did show an increased sensitivity to the neurotoxin MPTP (See Section 3.1). The *pink1* knock down fish, when exposed to MPTP, swam significantly less and experienced a greater loss of tyrosine hydroxylase (TH)-immunoreactive neurons than controls exposed to MPTP alone. TH is the rate limiting enzyme in the synthetic pathway for dopamine and often used to identify dopaminergic cells.

A third MO knock down of *pink1* also found little loss of dopaminergic neurons but did note some abnormal morphology of said neurons (Xi et al., 2010). The *pink1* deficient fish exhibited altered locomotor activity, including decreased swimming behaviour and response to touch. There was also decreased mitochondrial function due to reduced numbers of mitochondria and a loss of cristae (Xi et al., 2010, 2011).

A *pink1* ^−/−^ mutant zebrafish, identified using TILLING, showed loss of dopaminergic neurons, increased mitochondrial size and decreased mitochondrial Complex I and III activity in both larvae and adults (Flinn et al., 2013). These fish also upregulated the apoptosis regulator *TigarB* (orthologue to human *TIGAR*), and an MO knock down of *TigarB* completely rescued dopaminergic neuron loss in the *pink1* mutants. Flinn et al. (2013) verified the results by creating first a MO knock down of *pink1,* which mirrored the mutant results, and then a double knock down of *pink1* and *TigarB*, which rescued the dopaminergic neurons and mitochondrial function.

The many contradictory results from knock downs of *pink1* in zebrafish are suggestive of off-target effects, stemming from the different MOs used (See Section 5: Discussion). The mutant model presented by Flinn et al. (2013) may present a more stable platform to study the function of *pink1*. The congruence between the findings of their MO knock down and mutant models is strong evidence of a successful MO knock down. This study also indicated that *pink1* plays an important role in dopaminergic neuron development and mitochondrial function and suggests a target (*TigarB*) for future therapeutic intervention.

### 2.4 DJ-1

DJ-1 is a redox-sensitive chaperone that protects against oxidative stress. Mutations in the gene encoding DJ-1 (also known as PARK7) have been linked to autosomal-recessive EOPD, causing altered mitochondrial morphology and increased production of ROS (Cookson, 2005). *DJ-1* null mice showed no apparent direct effects in dopaminergic neurons, but those neurons were more sensitive to oxidative stress (Kim et al., 2005). *Drosophila* studies of an interference RNA knock down of *DJ-1* showed degrees of dopaminergic neuron loss and an increased sensitivity to oxidative stress (Meulener et al., 2005; Yang et al., 2005).

Zebrafish dj-1 is 83% identical to the human version and is expressed throughout the brain, including in dopaminergic neurons in the CNS (specifically cell groups in the olfactory bulbs, diencephalon and telencephalon) (Bai et al., 2006).

An immunohistochemical study of a *dj-1* MO knock down showed no decrease in the numbers of dopaminergic (TH-positive) neurons. As in the mouse model, the zebrafish dopaminergic neurons were more susceptible to apoptosis and showed increased sensitivity to hydrogen peroxide or to a proteasome inhibitor. In zebrafish subjected to oxidative stress, *dj-1* is upregulated. This indicates that mutations in *dj-1* may impair the response of dopaminergic neurons to environmental stress, leaving them susceptible to cell death (Baulac et al., 2009).

Recently, the CRISPR-Cas9 method was used to produce a dj-1 deficient zebrafish, which developed normally until the adult stage, at which point the fish began to exhibit low body mass and lower levels of TH. A proteomic analysis on the brains revealed a dysregulation in proteins involved with mitochondrial mitophagy, redox regulation, stress response and inflammation (Edson et al., 2019).

As only one knock down study of *dj-1* was characterized in zebrafish, it is difficult to assess its effectiveness (Baulac et al., 2009). The CRISPR study is very promising as the fish can be studied in adulthood, compared to the MO knock downs that could only be achieved in larvae. In general, the results mirror those from the mice study, and therefore the gene appears to have a similar function to its mammalian counterpart and may provide a good basis for future study.

### 2.5 LRRK2

Mutations in the gene encoding LRRK2 (Leucine-rich repeat kinase 2) are the most prevalent cause of autosomal dominant PD in humans (Blandini and Armentero, 2012). Not much is known about the function of LRRK2*,* but it seems to be involved in neurodegeneration and kidney function (MacLeod et al., 2006). There are contradictory results with *Drosophila* models of *LRRK2* deficiency. One study reports reduction of dopaminergic neuron numbers (Lee et al., 2007), but the other saw no change in the neurons (Wang et al., 2008). In mice, disruption of *LRRK2* had no obvious effect on dopaminergic neurons and no increased sensitivity to the neurotoxin, MPTP (Andres-Mateos et al., 2009).

The zebrafish orthologue lrrk2 has a 38% similarity to its human counterpart (Sager et al., 2010). An MO knock down of *lrrk2* caused severe morphological defects, a loss of diencephalic TH-positive cells and death by 3 dpf (Sheng et al., 2010). Due to the severe phenotype, another MO knock down was created targeting a domain of *LRRK2* called WD40, which is associated with PD-inducing mutations in humans. These fish showed increasing loss of diencephalic dopaminergic neurons with increasing MO concentration. The WD40 knock downs also had reduced swimming activity that could be rescued by wild-type zebrafish or human LRRK2 or L-DOPA, a common treatment for the symptoms of PD (Cools, 2006; Sheng et al., 2010).

A replication of the study by Sheng et al. (2010), however, showed no dopaminergic neuron loss in a knock down of the WD40 domain of *LRRK2* (Ren et al., 2011). An analysis of swimming behaviour showed no differences between the knock downs and controls.

A recent study developed a knock down which reduced the levels of lrrk2 by 50%, and showed a reduction in the numbers of dopaminergic neurons and moderate defects in body morphology (Prabhudesai et al., 2016). The knock down also upregulated other PD-associated genes and caused β-synuclein (see below) aggregates in the diencephalon, midbrain and hindbrain.

There is some evidence to suggest that loss of *lrrk2* function is associated with dopaminergic neuron loss, but results present too much variation to be definitive. Again, such findings highlight the variety of results from MO knock downs. A direct replication of Sheng et al. (2010) failed to achieve the same outcomes. Ren et al. (2011) suggested the recreation of the knock down using ZFNs; this or CRISPR would help to clarify the function of *lrrk2* in zebrafish.

### 2.6 Alpha-Synuclein

Lewy bodies made of insoluble α-synuclein aggregates are a feature of PD and are thought to be caused by the dysregulation of α-synuclein, which is produced by the gene *SNCA* (Spillantini et al., 1998). In addition to α-synuclein, mammals also have β- and γ-synucleins, each encoded by its own gene (Clayton and George, 1998). In zebrafish, there is no evidence of a gene encoding α-synuclein. This gene may have been lost over time because of the overlapping function between the other synucleins (Chen et al., 2009); zebrafish do possess β-synuclein and co-orthologues for γ-synuclein (γ_1_ and γ_2_), but only β and γ_1_ are expressed in the CNS (Sun and Gitler, 2008).

An MO knock down of β- or γ_1_-synuclein caused decreased motor activity; and knock down of both β and γ_1_ caused delayed differentiation of dopaminergic neurons, reduced dopamine levels and impaired motor functions. Interestingly, expression of human α-synuclein can rescue this phenotype in zebrafish (Milanese et al., 2012).

Using a DNA plasmid, researchers created a zebrafish model that expresses large amounts of human α-synuclein, which resulted in high mortality rates but also α-synuclein aggregates and α-synuclein-induced apoptosis. This severe phenotype was greatly improved by the addition of CLR01, a “molecular tweezer” known to inhibit the assembly and toxicity of many amyloidogenic proteins (Prabhudesai et al., 2012).

Additionally when γ_1_-synuclein was overexpressed, fish developed aggregates within neurons similar to those found within fish expressing human α-synuclein (O’Donnell et al., 2014; Lulla et al., 2016). Zebrafish larvae were exposed to ziram, a fungicide that increases α-synuclein expression in rat cells. The larvae showed neuronal aggregates and dopaminergic neuron toxicity, which could be prevented when treated with CLR01.

In summary, studies suggest that although zebrafish do not possess an α-synuclein orthologue, γ_1_-synuclein may perform a comparable function to human α-synuclein, however more research is required to confirm this hypothesis. The human α-synuclein gene can be successfully expressed in zebrafish and causes aggregates within neurons. Studies also confirmed the successful use of CLR01, a potential PD therapy, in zebrafish.

## 3 Chemical-Based Models of Parkinson’s Disease

For a summary of these models refer to Table 2

**TABLE 2:** Chemical-based zebrafish models of Parkinson’s Disease.

Chemical	Adults/Larvae	Studies	Results
MPTP	Adults/Larvae	Bretaud et al. (2004), Lam et al. (2005), McKinley et al. (2005), Sallinen et al. (2009)	Loss of DNs in diencephalon, decreases in swimming responses
	Adults	Anichtchik et al. (2004)	Intracerebral/intramuscular injections cause locomotor defects and decreased dopamine levels
	Larvae	Wen et al. (2008)	GFP line to visualize TH-positive neurons. Loss of neurons in posterior and the hypothalamus
	Larvae	Dukes et al. (2016)	GFP line to visualize mitochondria in DNs. Reduction of mitochondrial transport
	Adults	Babu et al. (2016)	Injection. Increased synuclein, identified 73 proteins upregulated with MPTP exposure
6-OHDA	Adults	Anichtchik et al. (2004)	Intracerebral/intramuscular injections cause locomotor defects and decreased dopamine levels
	Larvae	Parng et al. (2007)	Reduction TH-positive neurons in hypothalamus, posterior tuberculum, ventral thalamus and pretectum
	Larvae	Feng et al. (2014)	Minocycline, Vitamin E and Sinemet can rescue locomotor defects
	Adults	Vijayanathan et al. (2017)	Decreased TH-positive neurons, decreased swim speed, spontaneous recovery by 30 dpf
Rotenone	Adults	Bretaud et al. (2004)	High doses: lethality, low doses: no morphological or locomotor defects
	Adults	Wang et al. (2017)	Decrease in DA and TH expression, behavioural abnormality, olfactory deficits
Paraquat	Adults	Bortolotto et al. (2014)	Injection. Learning and motor deficits, increase in DA
	Larvae	Nellore and Nandita, (2015)	Morphological defects, increased apoptosis
	Adults	Wang et al. (2016)	Upregulation of antioxidant genes
	Adults	Nunes et al. (2017)	Abnormal behaviour, increase in aggression
	Adults	Müller et al. (2018)	Injection. Learning and motor deficits, Sodium selenite diet prevents motor symptoms
	Larvae	Wang et al. (2018)	Locomotor defects, reduced mitochondrial activity
Titanium dioxide nanoparticles	Larvae	Hu et al. (2017)	Loss of DNs, increase PD-associated gene expression, increase in ROS

*DNs, Dopaminergic neurons DA - Dopamine TH, Tyrosine hydroxylase GFP, Green fluorescent protein ROS, reactive oxygen species.

### 3.1 MPTP

Administration of 1-methyl-4-phenyl-1,2,3,6-tetrahydropyridine (MPTP) has been a well-documented method of inducing dopaminergic neurodegeneration in a variety of animals (Dauer and Przedborski, 2003). The highly lipophilic MPTP crosses the blood brain barrier and is converted to the metabolite 1-methyl-4phenylpyridinium ion (MPP+) by monoamine oxidase B (Blandini and Armentero 2012). MPP+ has a high affinity for the dopamine transporter and is carried into the dopaminergic neurons of the substantia nigra where it blocks mitochondrial Complex I activity (Blandini and Armentero, 2012). It produces Parkinsonian-like symptoms in humans; however, it does not create the Lewy body-like inclusions that are present in PD (Blandini and Armentero, 2012). Exposure of *C. elegans* to MPTP causes reduced movement and degeneration of dopaminergic neurons (Braungart et al., 2004). Similarly, MPTP exposure in *Drosophila* causes oxidative stress and inflammation as well as behavioural deficits (Abolaji et al., 2018; Aryal and Lee, 2019). Rats injected with MPTP do not develop Parkinsonian-like symptoms, for unknown reasons (Beal, 2001). To achieve dopaminergic depletion in mice, large doses of MPTP must be administered regularly. Injecting MPTP in conjunction with probenecid prevents the clearance of MPTP from the brain and kidneys, such that a chronic depletion of dopamine may be realised (Petroske et al., 2001; Meredith et al., 2008).

When exposed to MPTP, larval zebrafish exhibited a 39% loss of dopaminergic neurons in the ventral diencephalon (Lam et al., 2005; Kalyn et al., 2020). Both larval and adult zebrafish showed decreases in swimming responses after treatment with MPTP (Bretaud et al., 2004; Sallinen et al., 2009). Inhibition of monoamine oxidase B or the dopamine transporter mitigates the neuronal loss following administration of MPTP, which confirms the same mechanism of action as in mammals (Lam et al., 2005; McKinley et al., 2005).

A transgenic zebrafish, expressing green fluorescing protein (GFP) in neurons expressing vesicular monoamine transporter 2, was created to characterize the loss of TH-positive neurons within the context of all monoaminergic neurons present in the nervous system (Wen et al., 2008). They noted loss of TH-positive neurons in the posterior tuberculum of the ventral diencephalon and the hypothalamus following MPTP exposure. Another transgenic GFP line was developed to visualize the mitochondria of dopaminergic neurons (Dukes et al., 2016). After exposure to MPP+, there was a reduction of all mitochondrial transport, which likely plays a role in the dopaminergic neuron loss.

A study on adult zebrafish injected intraperitoneally with MPTP showed a decrease in swimming behaviour and increased “freezing,” as a response to stress, but also increased γ_1_-and γ_2_-synuclein expression, which was visualized with an antibody against human α-synuclein (Babu et al., 2016). This study was mainly proteomic in nature and identified 73 proteins that demonstrated altered expression in the MPTP-induced state.

In summary, one of the most popular chemical models of PD, MPTP and its metabolite (MPP+) cause reliable dopaminergic neuron loss in both larval and adult zebrafish and the dosages for both are well established. Like most chemical models, this one does not cause Lewy body-like inclusions in the brain. The use of transgenic GFP lines make the characterization of neuron loss and intracellular activities easy to visualize *in vivo*. In addition, Babu et al. (2016) identified new, potential targets for study when they identified proteins that have altered expression after MPTP exposure.

### 3.2 6–Hydroxydopamine

The hydroxylated analogue of dopamine, 6-hydroxydopamine (6-OHDA), was used to create one of the first animal models of PD (Schober, 2004). It has a high affinity for the dopamine transporter, which carries 6-OHDA inside dopaminergic neurons where it accumulates and causes cell death (Blandini and Armentero, 2012). In mice and rats, administration of 6-OHDA caused reduction in dopaminergic neuron numbers and marked locomotor defects but no evidence of Lewy body-like inclusions (Schober, 2004; Stott and Barker, 2014). *C. elegans* exposed to 6-OHDA showed selective degeneration of dopaminergic neurons (Nass et al., 2002).

Intramuscular injections of 6-OHDA in zebrafish caused locomotor defects and decreased dopamine levels which indicate that 6-OHDA crosses the blood-brain barrier (BBB) more readily in zebrafish than in mammals (Murray et al., 1975; Anichtchik et al., 2004). When 6-OHDA was administered to larval zebrafish there was increased oxidation throughout the brain and a significant reduction in the number of TH-positive neurons in the diencephalon (hypothalamus, posterior tuberculum, ventral thalamus, and pretectum) (Parng et al., 2007). Another study of larval fish found that the antioxidant Vitamin E was able to rescue the locomotor defects and the decrease in TH expression caused by 6-OHDA exposure (Feng et al., 2014). They also found that the microglia inhibitor, minocycline, can reverse both locomotor defects and the expression of inflammatory genes in 6-OHDA-treated fish. Feng et al. (2014) also assessed a clinical PD treatment, Sinemet (a combination of L-dopa and carbidopa), and found that it can also rescue locomotor activity and reduced the expression of *pink1* and *parkin* in treated animals.

A more recent study injected 6-OHDA directly into the diencephalon and demonstrated both decreases in TH immunoreactive neurons and decreases in swim speed and distance travelled of the fish (Vijayanathan et al., 2017). Interestingly, animals showed recovery of the lost TH immunoreactive neurons 30 days after injection.

The effects of 6-OHDA have been well characterized in both adult and larval zebrafish, and although the toxin does not induce Lewy bodies found in PD, it seems to be a good model for screening therapeutic options for PD. There is a suggestion that 6-OHDA has greater effects on the brain of zebrafish than in rodents because of the ease with which it crosses the blood-brain barrier (Murray et al., 1975). Vijayanathan et al. (2017) found that the fish recover from 6-OHDA spontaneously. This may indicate that neurogenesis is occurring, which is common in zebrafish (Kizil et al., 2012).

### 3.3 Rotenone

Rotenone is widely used as both an insecticide and piscicide (fish toxin). It readily crosses the blood brain barrier and is a high-affinity inhibitor of Complex I of the mitochondrial ETC (Sherer et al., 2003; Bové et al., 2005). Humans exposed to high levels of rotenone are 2.5 times more likely to develop Parkinsonian-like symptoms than the general population (Innos and Hickey, 2021). In rats, rotenone exposure causes degradation of the nigrostriatal dopaminergic neurons and behavioural symptoms characteristic of PD (Sherer et al., 2003). The affected neurons possess intracellular inclusions that appear to be Lewy bodies, showing immunoreactivity for α-synuclein and ubiquitin as with human Lewy bodies (Blesa and Przedborski, 2014). In *Drosophila* and *C. elegans*, rotenone causes locomotor deficits and loss of dopaminergic neurons (Coulom and Birman, 2004; Zhou et al., 2013). Chronic oral administration of rotenone to mice resulted in behavioural impairments and a significant loss of TH-positive neurons. In addition α-synuclein expression was increased in remaining neurons as the treatment progressed (Inden et al., 2011).

Exposure of larval zebrafish to rotenone at LD_50_ concentration (50 nM) resulted in moderate locomotor defects and 36% reduction in the amount dopaminergic neurons in the ventral diencephalon (Kalyn et al., 2020). A study of environmental neurotoxins found that high dosages of rotenone were lethal to adult zebrafish after a few days, and embryos exposed to high dosages expressed increased pigmentation and eventually death (Bretaud et al., 2004). However, lower doses caused no morphological or locomotor defects.

In another study, adult zebrafish administered rotenone (chronic exposure over 4 weeks) exhibited both motor and non-motor (cognitive) effects of PD which mirrors the effects of the Kalyn et al. (2020) study in larvae (Wang et al., 2017). This contradicts the findings of Bretaud et al. (2004), which showed no locomotor defects after 4 weeks of exposure to the same dosage of rotenone (2 μg/L). In the Wang et al. (2017) study, rotenone-treated fish showed a reduction in dopamine concentrations in the brain as well as a decrease in TH expression. They also exhibited a 70% reduction in swimming duration and distance travelled, which may be comparable to the bradykinesia observed in PD patients. In a light/dark box test, treated fish spent more time in the light and showed a longer latency to enter the dark component, which Wang et al. (2017) suggests indicates depression-like behaviour. Treated fish also showed a decreased affinity for amino acids, which may indicate a loss of olfactory function, a common symptom in PD (Ruan et al., 2012). This study also examined the expression of PD-related genes and found that in treated fish, *dj-1* was down-regulated by 60% and *lrrk2* was up-regulated. Other genes, *synuclein* (unspecified), *parkin* and *pink1* remained unchanged.

The chronic exposure of zebrafish to rotenone and the development of clinical and biochemical symptoms common to PD make this an interesting model to study the disease. There is some contention about dosage and reported symptoms. The role of rotenone as an aquatic pesticide means only small dosages can be used in any fish model. Rotenone also causes Lewy body-like inclusions in rodents, although it is unclear if that is the case in zebrafish.

### 3.4 Paraquat

Paraquat or N,N′-dimethyl-4-4′-bipiridinium is a widely used agricultural herbicide that subsequently becomes an aquatic contaminant due to run-off (Dinis-Oliveira et al., 2006). It is thought that paraquat impairs mitochondrial Complex I, leading to problems with the ETC and consequently the overproduction of ROS (Wang et al., 2018). It is very similar in structure to the MPTP metabolite MPP+ and also has specificity for the same dopamine transporter, and therefore it acts mainly on dopaminergic neurons (Manning-Bog et al., 2003; Bové et al., 2005). Like rotenone, humans exposed to high levels of paraquat, usually during the manufacture or use of the herbicide, develop Parkinsonian-like symptoms (Boyd et al., 2020). Chronic paraquat exposure in rats caused an increase in anxiety-like behaviour, a loss of olfactory function and a marked decrease in mitochondrial function (Czerniczyniec et al., 2011). In *Drosophila and C. elegans,* paraquat exposure caused severe locomotor deficits (Jahromi et al., 2015; Bora et al., 2021).

An early study of paraquat found that chronic water exposure did not cause any motor defects or changes in TH levels of adult or larval zebrafish (Bretaud et al., 2004). However, subsequent studies report that paraquat-exposed larval fish showed increased oxidative stress, upregulation of antioxidant genes and general apoptosis (Nellore and Nandita, 2015; Wang et al., 2016). Mitochondrial respiration was reduced by 70% in treated fish (Wang et al., 2018). At LD50 concentrations, paraquat did not cause locomotor defects but did cause a 16% reduction in the number of dopaminergic neurons in the ventral diencephalon of larval zebrafish (Kalyn et al., 2020).

However, adult zebrafish injected intraperitoneally with paraquat showed locomotor defects, increased “freezing” behaviour indicative of anxiety and increased aggression when presented with their own reflection (Nunes et al., 2017; Müller et al., 2018). The fish experienced some cognitive deficiencies, as they could not learn associations in a Y-maze as well as controls. There was no decrease in dopamine levels, but depending on the paraquat dosage, dopamine transporter (DAT) expression decreased (Bortolotto et al., 2014). Sodium selenite (Na_2_SeO_3_), an antioxidant, rescues some of the behavioural symptoms (Müller et al., 2018).

As with the other chemical models, there seems to be a disagreement with effective dosages to induce PD-like symptoms. Like rotenone, paraquat does not seem to cause Lewy body-like inclusions in zebrafish, but some of the later studies seem to have developed stable models to investigate the underlying mechanisms and potential therapeutic substances.

### 3.5 Titanium Dioxide Nanoparticles

Titanium dioxide nanoparticles are widely produced for various commercial applications. Unfortunately, they can also cause health issues due to environmental or workplace exposure (Shi et al., 2013). Large scale exposure of mice to titanium dioxide nanoparticles caused motor deficits and reduction of dopaminergic neurons in the midbrain substantia nigra (Heidari et al., 2019). When embryonic zebrafish were exposed to titanium dioxide nanoparticles from 0–96 hpf, the titanium accumulated in the brain, causing the generation of ROS and ultimately cell death in the hypothalamus. The nanoparticles also caused loss of dopaminergic neurons and increased the expression of *pink1* and *parkin* (Hu et al., 2017). This shows promise as a method of inducing a Parkinsonian-like state, and future work could confirm its validity.

## 4 Chemogenetic Ablations

These models are unique in that they employ a combination of genetic and chemical methods to achieve the ablation of specific cells. The models discussed in this review use nitroreductase (NTR), a bacterial enzyme derived from *Escherichia coli*, which converts the antibiotic metronidazole into a cytotoxic metabolite (Curado et al., 2008). By creating a transgenic zebrafish line that expresses NTR under a specific promoter, metronidazole will only be converted into a toxin in those cells where NTR is present and when metronidazole is administered. This leads to spatially and temporally specific ablation of targeted cells (Pisharath and Parsons, 2009).

### 4.1 Cytotoxic Metabolite of Metronidazole

A study created a transgenic zebrafish expressing NTR fused to cyan fluorescent protein (CFP) under the control of the dopamine transporter (DAT), in the telencephalon, diencephalon, olfactory bulb and caudal hypothalamus (Godoy et al., 2015). Administration of the anti-bacterial pro-drug metronidazole at 1 dpf resulted in loss of DAT-expressing neurons at 5 dpf in addition to motor impairments. Some motor function was re-established by 7 dpf when there was evidence of new DAT-expressing cells; however, there was still a deficit at 14 dpf. A follow-up study in adult transgenic zebrafish found that a 24 h exposure to metronidazole resulted in significant loss of dopaminergic neurons in the olfactory bulb and a decrease in the ability to smell (Godoy et al., 2020). Another study has suggested that using a similar drug, ronidazole, can achieve comparable results at lower concentrations (Lai et al., 2021). The cytotoxic metabolites of these drugs appear to induce Parkinsonian-like symptoms, like other chemical models, and may provide a basis for understanding mechanisms underlying neuronal loss.

## 5 Discussion

As discussed throughout this review zebrafish present numerous promising avenues for research. They generally possess the same genes as humans with comparable functions and most of the toxins mentioned produce similar outcomes. This provides an outstanding opportunity to study basic underlying mechanisms in a more tractable model than offer by traditional mammalian species. Advanced imaging techniques in conjunction with genetic manipulation make zebrafish a highly versatile model for researching PD. The specific aspects of each model were reviewed in detail in Sections 2, 3, 4. In this section, the strengths and weaknesses of models are discussed. For instance, many orthologues of PD-related genes have been identified in zebrafish and evidence suggests that fish can provide useful insights into both their normal functions and their roles in disease progression. However, the major issue with many of the gene-based models of PD is their current reliance on MO antisense oligonucleotides to create gene knock downs. It became evident during the evaluation of these models that there was great variation in findings between different studies, even when targeting the same gene. For example, three different knock downs of *pink1* had vastly different results (Anichtchik et al., 2008; Sallinen et al., 2010; Xi et al., 2010). In another case, a direct replication of an *lrrk2* knock down was unable to reproduce the same results, even when using the same methods (Ren et al., 2011). This lack of reproducibility obscures the function of these genes in zebrafish because it is unclear whether the knock downs have off target effects. It has been well-documented that MOs can often have severe off-target effects, and it is difficult to differentiate between specific and non-specific effects (Schulte-Merker and Stainier, 2014; Blum et al., 2015). Some researchers, therefore, suggest that MOs should not be used as a primary method of studying gene function (Kok et al., 2015), but others insist that they are still valuable if the proper controls are in place. Eisen and Smith (2008) outlined guidelines for using MOs appropriately and avoiding off-target effects. While some of the reviewed studies employed one or more of these techniques, few followed all of the recommended guidelines. Consequently, the relative accuracy of these studies cannot be directly compared, except in cases where they were employed alongside a strain made by another technique as accomplished by Flinn et al. (2013) (See Section 2.3). Otherwise, the accuracy of models that utilise MOs may be compromised. The CRISPR/Cas9 system however, shows immense promise for the creation of genetically altered zebrafish lines and this genomic editing technique presents an opportunity to create more accurate gene knock downs/ins, which may elucidate some of the gene functions that are unclear in other models. It also enables researchers to examine gene knock downs in adult animals, as MOs generally only work until the larvae is a few days old. The study by Edson et al. (2019), created a *dj-1* knock down with CRISPR that successfully grew to adulthood.

In addition to evaluating the methods of creating genetic models, a critical review of the literature must also consider the unique characteristics of zebrafish. A possibly problematic feature of zebrafish is their duplicated genome, when compared to mammals. As mentioned in the reviews of gene-based models, zebrafish may have one or two homologous genes corresponding to a single human gene (Lieschke and Currie, 2007). Unfortunately, there is no evidence of consistency in the duplicated gene functions. The doubled genes may have unrelated functions, or alternatively, they may have semi-overlapping functions, which may be exploited to provide insight into different aspects of the genes’ functions by selectively knocking out one or the other (Newman et al., 2014).

Another feature of the zebrafish that sets it apart from mammals and that is relevant to both gene-based and chemically-induced models is the high level of post-embryonic neurogenesis that persists into adulthood in the fish. This is potentially problematic in studies of neuronal loss, as recovery from these states is very possible in zebrafish, unlike in mammals where recovery of neurons it is greatly limited (Kizil et al., 2012).

A further issue with current genetic models of PD in zebrafish is that PD is usually a disease of adults or older individuals. However, most of the models discussed in this review were performed on larvae, juvenile or young adult zebrafish. In fact, MOs are only effective when injected in the single cell stage and only function for a few days, therefore serious questions arise as to how well any such model can represent the disease as a whole (Eisen and Smith, 2008). With the advent of new genetic manipulation techniques mentioned above, it is possible to generate animals that express these PD phenotypes into adulthood. Generally, housing animals into senescence can become expensive and add years to potential studies, however the cost can be mitigated with economical animals like zebrafish.

Another general problem with attempts to replicate PD in any animals is that they do not naturally develop the full array of symptoms associated with the disease in humans. The genetic models can elicit some of the features of PD, but they never precisely replicate it. The chemical models generally do not induce PD, but instead use different mechanisms to achieve similar phenotypes. These mechanisms do not necessarily represent all aspects of the disease accurately. For instance, most of the chemical models of PD successfully induce a Parkinsonian-like state in zebrafish with losses of dopaminergic neurons and some behavioural symptoms, but none of them recreate the Lewy body-like inclusions seen in humans with the disease.

A final common issue with the reviewed models is that there is very little replication of experiments. Compared to other medical models, zebrafish are relatively new on the scene. Therefore, many of these models have only been the subject of one or two studies to date, with the notable exception of a handful of well-established models induced by chemicals such as MPTP. In contrast, there are many transgenic mouse models of PD (Duty and Jenner, 2011; Sharma et al., 2017). In time, further replication of previous studies may serve to solidify the status of these models and provide a basis to develop more complex models.

Despite some of the issues that one must consider when developing a zebrafish model of PD, there are several distinct advantages. As mentioned above, zebrafish are relatively easy to manipulate genetically, and a wide variety of custom strains can be created to allow sophisticated imaging techniques to be performed. With the CRISPR/Cas9 system, gene knock downs can be achieved in adult animals to examine this typically adult disease in older fish. This genetic adaptability can also be applied in conjunction with other areas of research, such as behavioural screens and high-throughput screens.

### 5.1 Future Directions

#### 5.1.1 Behavioural Screens

A practical method of assessing disease model effects on a normal phenotype is to examine changes in behaviour (Khan et al., 2017). For rodent models, there is a wide range of paradigms available to examine the effects of experimental treatment on behaviour, ranging from simple evaluation of locomotion to complex cognitive abilities like learning and memory (Sousa et al., 2006). Accordingly, rodent behavioural paradigms have been used extensively to study PD (Ameen-Ali et al., 2017; Vingill et al., 2018).

Zebrafish also possess a wide repertoire with many normal and abnormal behaviours catalogued (Kalueff et al., 2013). Zebrafish are inherently communal animals, living in shoals, so they display a wide variety of social behaviours. They also have well-documented expressions of fear and anxiety, and they can learn complex associations (Lieschke and Currie, 2007). Consequently, there have been many behavioural tests developed for zebrafish that are suitable for models of PD.

Several zebrafish paradigms are analogous to well established ones developed for rodents (Champagne et al., 2010). For instance, the novel tank test is an assessment of anxiety-like behaviour in zebrafish to a new stark environment and can be compared to the open-field test in rodents (Blaser and Rosemberg, 2012; Harro, 2018). There are also numerous paradigms that test cognitive behaviours, as the fish can rapidly learn associations using various unconditioned stimuli (Karnik and Gerlai, 2012). Zebrafish are highly visual animals and can differentiate between basic colours or patterns, but they can also form associations using olfactory or auditory stimuli (Colwill et al., 2005; Braubach et al., 2009; Avdesh et al., 2012; Doyle et al., 2017). Many common apparatuses used for studying cognitive behaviours in rodents have been adapted for zebrafish, including shuttle boxes, Y-mazes, T-mazes, and plus-mazes (Pather and Gerlai, 2009; Gerlai, 2010; Sison and Gerlai, 2010). Zebrafish training is highly reproducible and can therefore be automated and performed using groups of animals (Wyeth et al., 2011; Miller and Gerlai, 2012; Doyle et al., 2017). Commercially-available software offers three-dimensional, automated tracking of multiple fish simultaneously (Stewart et al., 2015).

Most of these learning paradigms are suitable for use with adult zebrafish; however, only the simplest behavioural assays are suitable for larvae (Colwill and Creton, 2011; Kalueff et al., 2013). Larvae can be examined for general behavioural abnormalities, but larvae under 5 dpf do not swim well (Lindsey et al., 2010). By 30 dpf, however, juvenile fish can perform in adult-appropriate paradigms (Kalueff et al., 2013; Merovitch, 2016).

In summary, many of the models reviewed in this study use some form of behavioural analysis to examine different aspects of PD. Several experiments use larvae, which limits the available behavioural analysis paradigms; however, the use of genetically altered zebrafish that can survive to adulthood in a disease-like state, presents the opportunity to examine disease models in older animals. Chemical-based models utilising adults are also viable and have been studied with behavioural paradigms, as reviewed in Section 3. Adult animals display more complex cognitive abilities than larvae, which may be studied to provide greater insight into the cognitive deficits seen in PD. However, while some paradigms can be used with groups of animals, none of the reviewed models utilised large-scale behavioural tests. Adaptation of some of these paradigms to use larger numbers of fish would allow rapid and efficient assessment of cognitive or behavioural defects induced in the disease models.

### 5.1.2 High-Throughput Screening

One of the most touted benefits of the zebrafish is their suitability for high-throughput screening (Gerlai, 2010). Rodents are expensive and relatively large, which makes them less practical to use in drug screens. High throughput screens with *C. elegans* and *Drosophila* also exist, but as invertebrates, they are less similar to humans. These species also possess tough cuticles, which may present barriers to the diffusion of drugs (Wells, 1998; Manev et al., 2003). By contrast, chemicals can enter the larval zebrafish by diffusion from the surrounding water (Langheinrich, 2003). Dissolving chemicals into water is simpler and quicker than injecting each animal (Parng et al., 2002). Moreover, larval zebrafish are quite small and can subsist in only 200 µl of water, so they fit efficiently in a 96-well plate, and only small amounts of test chemicals are needed (Best and Alderton, 2008). Often, larval zebrafish can be dosed with microliters of solution, compared to milliliters that may be required for rodents. This allows a greater number of animals to be tested with the same quantity of drugs (Parng et al., 2002). However, the aquatic environment does present some challenges. The precise delivery of some chemicals in water can be problematic. There may be some difficulty determining dosage, and chemicals may not be soluble in water, although this can often be remedied via solvents (i.e. dimethyl sulfoxide) (Rubinstein, 2003; Maes et al., 2012).

A further advantage is that the characterization of drug effects can be much simpler in larval zebrafish than in rodents (Parng et al., 2002). As previously mentioned, their transparent bodies, combined with the availability of numerous fluorescent reporters, make target areas or cell types relatively easy to visualize (Vaz et al., 2018). Each zebrafish larva can be tracked, and commercial systems are available to facilitate automated tracking (Stewart et al., 2015). The zebrafish has therefore shown promise as a subject for high throughput screening of potential disease treatments or discovery of novel compounds (Langheinrich, 2003; Bowman and Zon, 2010; Stewart et al., 2014; Vaz et al., 2018). Many of the gene knock downs mentioned in this review created altered phenotypes; high-throughput screening of drugs may be used to readily identify treatments that rescue these phenotypes.

There are, however, limitations to these high-throughput screens. Although this review found several cases of human drugs having the same function in zebrafish, physiological differences mean that some drugs that are effective on zebrafish may not work on humans (Bowman and Zon, 2010). Even in cases where drugs have the same function on the molecular level, they may interact differently within the context of human physiology (Van Dam and De Deyn, 2011). Most screens utilise chemical libraries of previously approved drugs, with the goal of finding new therapeutic applications for old drugs (North et al., 2007; Hao et al., 2010). These drugs have already been deemed safe for human use, so approval depends exclusively on effectiveness, thus streamlining the process. Larval zebrafish are well suited for these large-scale drug screens, but with the development of new genetically altered strains, there are increasing numbers of gene knock outs in adult fish that can be investigated (Kalueff et al., 2014). There have recently been increased efforts to develop large scale testing of adult fish. While adult fish do not have the same advantages as larvae in terms of water requirements, they do present opportunities for different types of behavioural screening.

As discussed in Section 5.1.1, several of the common behavioural paradigms are easily adaptable to accommodate large numbers of fish. One study presents a high volume study to identify fluorophores in adult zebrafish (Blackburn et al., 2011). This was performed in normally pigmented fish, but there are also strains of mutant zebrafish (e.g. CASPER) that remain transparent into adulthood, which would facilitate the visualization of fluorescence (White et al., 2008). The intersecting developments in genomic editing, high-throughput screening and behavioural screening collectively represent fertile ground for future research into zebrafish disease models of PD.

## 6 Conclusion

No animal model can perfectly mimic human disease, especially highly complex neurodegenerative diseases such as PD. However, the use of multiple animal models can mitigate their limitations. For such a nascent model organism, there is a surprising array of zebrafish models that investigate various aspects of PD.

Gene-based studies have established that zebrafish possess genes to that are homologous to human PD genes, and they generally serve similar functions. However, gene-based models that rely on MOs tend to show conflicting results, which detract from their value. Their use should follow strict guidelines to ensure repeatability, but off-target effects remain problematic regardless. Alternatively, new methods of genomic editing such as CRISPR show immense promise for more consistent generation of gene knock outs/ins. Whatever the method, researchers must account for the duplication within the zebrafish genome, which in some cases may even present promising avenues for future research.

Chemical methods can readily induce disease-like states but do not accurately model all aspects of the disease. There are several well-established chemically induced models of PD, which provide stable bases for study; and newer methods show great potential but are not currently as well studied. Zebrafish models typically show the same effects as the mammalian models when exposed to the same chemicals, indicating the validity of zebrafish for this application.

As a relatively new model organism, the zebrafish initially lacked the well-established body of research that surrounds some other animals, but with the advent of new genomic editing technology and the development of advanced imaging techniques zebrafish are swiftly becoming a highly favoured model. With the array of behavioural paradigms and high-throughput screens available, the zebrafish as a disease model is on the threshold of new discoveries.
